# From bench to bedside, 2-in-1 design expedites phase 2/3 oncology drug development

**DOI:** 10.3389/fonc.2023.1251672

**Published:** 2023-10-09

**Authors:** Cong Chen, Xuekui Zhang

**Affiliations:** ^1^ Biostatistics and Research Decision Sciences, Merck & Co., Inc., Rahway, NJ, United States; ^2^ Department of Mathematics and Statistics, University of Victoria, Victoria, BC, Canada

**Keywords:** Project FrontRunner, adaptive design, one-trial approach, accelerated approval, two-trial approach

By some accounts (1), it takes five oncology drugs an average of 6 to 7 years in clinical trials to yield one FDA approval, and behind it are 250 preclinical candidates out of 5,000–10,000 pre-discovery compounds. There are primarily three phases in oncology drug development, phase 1 for dose-finding, randomized-controlled phase 2 for clinical proof-of-concept of efficacy, and randomized-controlled phase 3 for confirmation of clinical benefits. In oncology, it is a routine practice to detect efficacy signals simultaneously in multiple tumor cohorts within the same trial or in a different one post-phase 1 dose-finding (2). Each cohort usually has a small sample size and is uncontrolled. Those promising tumor cohorts are often followed up with a phase 3 confirmatory trial, skipping the phase 2 trial. With the standard-of-care substantially improved in the last decade mainly due to the revolution of immune checkpoint inhibitors, this aggressive approach of skipping phase 2 to shorten the phase 2/3 development cycle is extremely risky and has already led to multiple high-profile setbacks.

To mitigate the phase 3 risk, sponsors must increase the sample size as well as the trial follow-up time to increase the study power, but this adds to the increasingly high cost (a typical phase 3 oncology trial in a first-line metastatic setting enrolls ~800–1,000 patients, takes ~3 years to complete, and costs ~$100 million). A futility analysis may be conducted to stop the trial early to limit the cost, but, in fear of making a wrong decision, the futility bar is often set too low and the analysis is too late. Traditional adaptive phase 2/3 designs allow the mid-trial adaptations of dose, population, and sample size but are less applied in practice mainly because of the perceived complexity and potential regulatory changes.

Unbeknown to the biostatistical community until more recently, when an adaptive phase 2/3 design is well planned, phase 2 data can be not only included in phase 3 analysis but also legitimately declared positive after a false No-Go decision to phase 3 (i.e., drug is active but the bar for a Go decision to phase 3 is set too high for phase 2 data to cross) without inflating the overall type I error (aka 2-in-1 design (3–5)). A false No-Go decision may be made when the follow-up is not long enough to fully manifest the treatment effect or when the bar is simply unrealistic. Unlike futility stopping in a phase 3 trial, a No-Go decision to phase 3 in a 2-in-1 design does not automatically mean a failed study. With the statistical rigor preserved, a positive outcome at the end of phase 2 of an adaptive phase 2/3 trial has the same merit as a standalone phase 2 trial. By mimicking a sequential phase 2/3 design (i.e., a separate phase 3 after a positive phase 2), the decision to phase 3 is based on a smaller sample size than in a mid-trial phase 3 futility analysis, making it possible to pause or stop the development program earlier to reduce unnecessary patient exposure in case of underwhelming treatment effect.

FDA has recently launched Project FrontRunner to encourage sponsors to use an earlier surrogate endpoint in a randomized-controlled trial to potentially support accelerated approval (AA) in an earlier treatment setting for advanced or metastatic disease (6, 7). This important initiative will likely have a profound impact on future drug development. The AA of oncology drugs is traditionally supported by single-arm trials. The emphasis on randomization and control fits the 2-in-1 design well because it is the default feature of any adaptive phase 2/3 trial. Project FrontRunner also proposed a one-trial approach to support both AA and regular approval (RA) in the same phase 3 trial, on top of the conventional two-trial approach of using phase 2 for AA and a separate phase 3 for RA, which fits the 2-in-1 design well, too (see **Figure 1** for the illustration). When the phase 2 trial is successfully expanded to Phase 3 after a preliminary analysis, it may follow the one-trial approach based on an interim analysis of phase 3 for AA and based on the final analysis for RA. In case of a false No-Go decision to expand, a positive phase 2 may still be considered for AA, while a separate phase 3 trial will be used for RA, following the two-trial approach. A statistically valid outcome is necessary for any regulatory approval. Even if AA is not granted at the end of phase 2 after a false No-Go decision to phase 3 due to strict type I error control, the positive outcome provides a solid footing for the next step. It is not a wasted effort by any means as a randomized-controlled phase 2 trial should have been conducted in the first place. This enhanced perspective may provide a much-needed incentive for a sponsor to apply the 2-in-1 design when the alternative options are deemed either too risky (straight phase 3) or too inefficient (sequential phase 2/3) (8).

**Figure 1:**
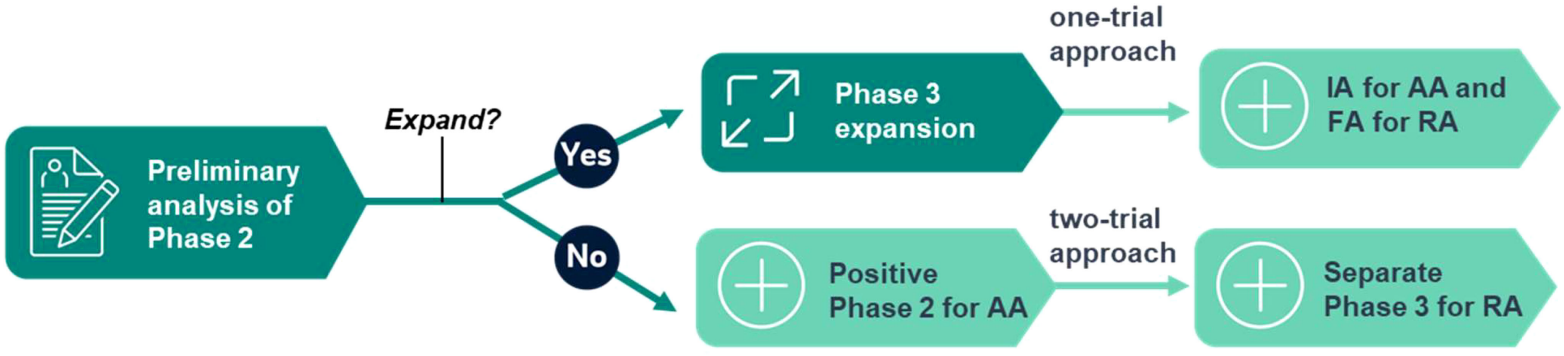
Fitting the 2-in-1 adaptive design into Project FrontRunner.

The 2-in-1 design concept is easily extendable to more than one path at multiple time points (4). On each path, a group sequential method may be applied to monitor the trial data and declare success early (9). The mid-trial adaptation of dose, population, and sample size can also be naturally incorporated into the design, which is an active research area in biostatistics. There will be indeed operational and statistical complexities with mid-trial adaptations. A practical advice is to only consider the design when there is no more than one critical issue to address in phase 2 (e.g., dose selection or biomarker enrichment). Otherwise, the sequential phase 2/3 approach is taken to resolve these issues before initiating the confirmatory trial. The separation between hypothesis generation and testing is a fundamental principle in scientific research. The 2-in-1 design implies that, when properly planned, not only can the data used for hypothesis generation be included in hypothesis testing but also it can also be formally tested. The consequence of this new philosophical realization has an impact on clinical research at different phases (10) and may also have some implications for preclinical research.

As one of the most significant breakthroughs in statistical design methodology in the last decade, the 2-in-1 design provides a powerful risk-mitigated cost-effective strategy in phase 2/3 oncology drug development. Since its inception 6 years ago, it has received tremendous attention from the oncology drug development community. A cross-industry and academia team of over 40 statisticians has formed a Drug Information Association working group to enhance its research, expand its influence, and broaden its areas of focus. This collaborative effort has resulted in the publication of more than 20 statistical papers that range from rigorous mathematical proofs to practical case studies and the delivery of numerous presentations.

## Author contributions

CC drafted the manuscript. All authors contributed to the article and approved the submitted version.
